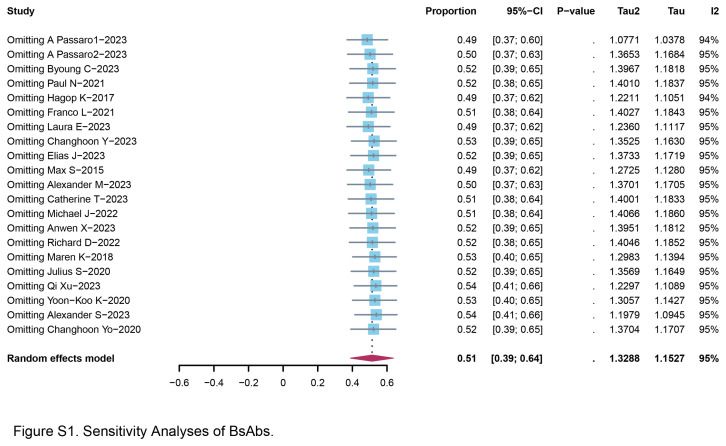# A comprehensive study of safety evaluation for novel bispecific antibodies combined with chemotherapy in cancer

**DOI:** 10.1097/JS9.0000000000001602

**Published:** 2024-05-17

**Authors:** Guo Lin, Xu Sun, Kai Kang, Ailin Zhao, Yijun Wu

**Affiliations:** a Division of Thoracic Tumor Multimodality Treatment, Cancer Center, West China Hospital, Sichuan University; b Laboratory of Clinical Cell Therapy, West China Hospital, Sichuan University; c Department of Hematology, West China Hospital, Sichuan University, Chengdu, Sichuan, People’s Republic of China

*Dear Editor*,

The achievements of immune therapy and targeted therapy highlighted the significance of antibodies, the research and development of antibodies is advanced gradually. Recent studies have emphasized bispecific antibodies (BsAbs) as an innovative modality within the dynamic landscape of cancer therapeutics^1^. The inherent advantage of BsAbs lies in their unique ability to engage dual sites, targeting both cancer cells and immune effectors concurrently^2^. This property orchestrates a precise and targeted immune response against tumors. This molecular interaction facilitates a selective destruction of cancerous cells while preserving the integrity of adjacent healthy tissue. Once engaged, the immune cells are activated, leading to the release of cytotoxic molecules and the initiation of an immune-mediated attack on the tumor cells. This mechanism not only capitalizes on the body’s natural defense mechanisms but also introduces a level of specificity that has the potential to revolutionize cancer therapy.

Kong *et al*.^3^ recently conducted a comprehensive systematic review and meta-analysis to presented toxicity profiles of PD-1/PD-L1 inhibitors and establish a clinically relevant landscape of adverse events of PD-1/PD-L1 inhibitors. The scope of BsAbs extends beyond hematologic malignancies to encompass solid tumors, marking a significant advancement in their therapeutic domain^4^. Preclinical studies serve as the foundational step in evaluating the safety prospects of BsAbs^5^. Despite the promising therapeutic potential, a comprehensive evaluation of safety parameters is imperative for the successful integration of BsAbs into clinical practice.

Therefore, we compared the treatment-related adverse events (tr-AEs) profiles of BsAbs, drawing from randomized clinical trials (RCTs) reported in the literature. Specifically, our assessment encompassed dual checkpoint blockade therapies targeting EGFR and cMet, PD-L1, and TGFβ, Gp100 and CD3, CD3, and CD19. A systematic search, inclusive of online databases and international conferences, was conducted up to January 2024, yielding a corpus of 273 articles. Following the exclusion of 25 duplicative studies, the titles and abstracts of 248 publications were meticulously scrutinized. A further exclusion of 85 studies lacking complete data and 121 non-RCT studies narrowed the selection to 42 articles for full-text review. Ultimately, 20 RCTs that met the inclusion criteria were identified and their flow depicted in Figure 1A. Seven out of the 20 studies^6–12^, with more than dual arms (excluding NCT04988295, which had three arms), were selected for Bayesian network meta-analysis. A comprehensive summary of study characteristics is presented in Table 1. The patient cohort across the 21 RCTs comprised 2401 individuals, encompassing seven distinct treatment regimens: chemotherapy, amivantama plus belazertinibe plus chemotherapy, amivantama plus chemotherapy, bintrafusp alfa, catumaxomab plus chemotherapy, Tebentafusp, and blinatumomab (Fig. 1B). Figure 1C suggests that BsAbs plus chemotherapy exhibited no greater toxicity than chemotherapy monotherapy, with no significant differences observed among various BsAbs. Additionally, Bayesian ranking profiles indicated that chemotherapy had a lower probability (32.2%) of encountering tr-AEs compared to other regimens, and blinatumomab emerged as the safest therapy with a probability of 15.6%. Consequently, our findings suggest no discernible toxicity disparities between BsAbs plus chemotherapy and single agent chemotherapy.

**Figure 1:**
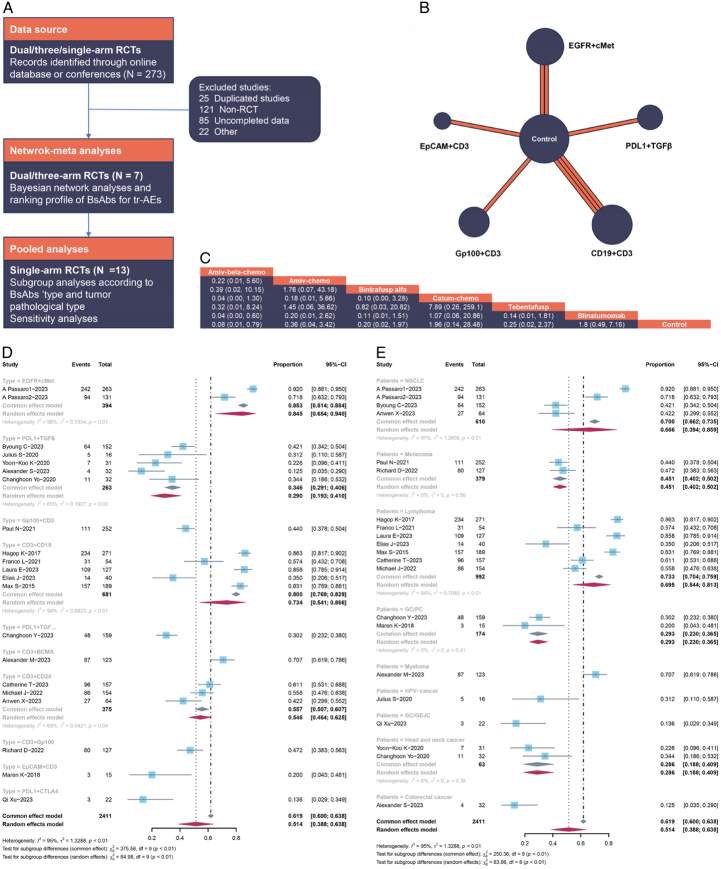
(A) Flowchart of study selection and analyses. (B) Network plot of seven treatments on tr-AEs. The width of lines is proportional to the number of trials, the circle is the sample size. (C) Multiple comparison for tr-SAE based on network consistency model (OR >1) indicates higher incidence rate of tr-SAE (Amiv-bela-chemo: amivantama plus belazertinibe plus chemotherapy. Amiv-chemo: amivantama plus chemotherapy. Catum-chemo: catumaxomab plus chemotherapy). Contorl including single agent pembrolizumab, ipilimumab, or chemotherapy. Forest Plot of incidence rate of tr-AEs, subgroup analysis according to (D) BsAbs’ type and (E) pathological type of cancer.

**Table 1:** Clinical characteristics of included clinical trials.

Study	Year	Phase	Pathology	Drug type	Sample size	Treatment
NCT04988295	2023	III	NSCLC^a^	EGFR+cMet	263 131 263	Amivantama plus belazertinibe plus chemotherapy Amivantama plus chemotherapy
NCT03631706	2023	III	NSCLC^a^	PDL1+TGFβ	152 152	Bintrafusp alfa Pembrolizumab
NCT01504256	2018	II	GC/PC^a^	EpCAM+CD3	15 16	Catumaxomab plus chemotherapy
NCT03070392	2021	III	Melanoma	Gp100+CD3	252 126	Tebentafusp Single agent pembrolizumab/ipilimumab/dacarbazine
NCT02013167	2017	III	ALL^a^	CD19+CD3	271 134	Blinatumomab Chemotherapy
NCT02393859	2021	III	ALL^a^	CD19+CD3	54 54	Blinatumomab Chemotherapy
NCT02101853	2023	III	ALL^a^	CD19+CD3	127 128	Blinatumomab Chemotherapy
NCT03427411	2020	II	HPV-associated cancer	PDL1+TGFβ	16	Bintrafusp alfa
NCT03833661	2023	II	Biliary tract cancer	PDL1+TGFβ	159	Bintrafusp alfa
NCT02699515	2020	I	Colorectal cancer	PDL1+TGFβ	31	Bintrafusp alfa
NCT02517398	2023	I	Colorectal cancer	PDL1+TGFβ	32	Bintrafusp alfa
NCT02699515	2020	I	Head and neck cancer	PDL1+TGFβ	32	Bintrafusp alfa
NCT03263572	2023	II	ALL^a^	CD3+CD19	40	Blinatumomab
NCT01466179	2015	II	ALL^a^	CD3+CD19	189	Blinatumomab
NCT04649359	2023	II	Myeloma	CD3+BCMA	123	Elranatamab
NCT03625037	2023	II	B-Cell Lymphoma	CD3+CD20	157	Epcoritamab
NCT03075696	2022	II	B-Cell Lymphoma	CD3+CD20	154	Glofitamab
NCT02570308	2022	II	Melanoma	CD3+Gp100	127	Tebentafusp-tebn
NCT03838848	2023	II	NSCLC^a^	PDL1+CTLA4	64	KN406
EMSO AISA	2023	-	GC/GEJC^a^	PDL1+CTLA4	22	Cadonilimab

a ALL, acute lymphoblastic leukemia; GC, gastric cancer; GEJC, gastroesophageal junction cancer; NSCLC, non-small cell lung cancer; PC, Peritoneal carcinomatosis.

In addition, supplementary analyses involved data from single-arm trials, encompassing a total of 13 trials^13–25^. These single-arm datasets involved 1146 patients who underwent treatment with BsAbs, and the reported incidence rate of tr-AEs is summarized. The pooled tr-AEs rate was calculated to be 51.4% (95% CI: 38.8–63.8%), with a statistically significant level of heterogeneity. A notable observation emerged from our analysis, revealing that the combination of PD-L1 and CTLA4 exhibited the lowest incidence rate of toxicity at 13.6% (95% CI: 2.9–34.9%) (Fig. 1D). Furthermore, colorectal cancer patients who received BsAbs demonstrated a favorable tr-AEs incidence rate of 12.5% (95% CI: 3.5–29.0%). Gastric cancer/peritoneal carcinomatosis and head/neck cancer also had the considerable safety, with no discernible evidence of significant heterogeneity (Fig. 1E). In order to ascertain the robustness of our findings and to mitigate the potential influence of individual trials on the overall outcomes, we conducted leave-1-out sensitivity analyses. This involved successively omitting each study from the analysis to assess the impact on the overall predictions. Figure S1 (Supplemental Digital Content 1, http://links.lww.com/JS9/C572) demonstrated that the overall predictions remained consistently stable across these sensitivity analyses. The omission of any individual study did not engender fluctuations in the tr-AEs incidence rate, affirming the reliability and resilience of our observed outcomes.

Our analysis revealed the tolerable incidence rate of tr-AEs associated with BsAbs (especially for colorectal cancer patients), compared with chemotherapy monotherapy. These findings augur well for the prospective clinical utilization of BsAbs. Nevertheless, it is imperative to acknowledge the intrinsic limitations stemming from the modest sample sizes and the inherent diversity of tumor types, contributing to an unavoidable heterogeneity. Despite the promising results from preclinical and early clinical studies, challenges exist in optimizing the safety profile of BsAbs. Cytokine release syndrome (CRS), a well-documented adverse event associated with immunotherapies, is one of the primary challenges. CRS is characterized by symptoms such as fever, hypotension, and flu-like symptoms and can pose a risk to patient safety. While CRS is generally reversible and manageable, its occurrence underscores the need for proactive monitoring and effective mitigation strategies. To address CRS and other potential safety concerns, researchers are actively engaged in refining the design and engineering of BsAbs. This includes modifications to the antibody structure, adjustments to dosing regimens, and the incorporation of novel technologies to enhance safety. For instance, the development of BsAbs with controlled Fc regions aims to modulate the immune response, potentially reducing the risk of CRS and other infusion-related reactions. Additionally, the identification of predictive biomarkers holds promise in tailoring BsAbs therapies to specific patient populations. Biomarkers associated with treatment response and adverse events can guide personalized treatment approaches, minimizing the risk of toxicity in patients who may be more susceptible to certain side effects. The integration of biomarker-driven strategies into clinical trials facilitates a more nuanced understanding of the safety profiles of BsAbs.

The dynamic landscape of ongoing research is pivotal in shaping the future of BsAbs in cancer therapy. Researchers are exploring various avenues to address current challenges and unlock the full therapeutic potential of these innovative molecules. One promising avenue of exploration involves the combination of BsAbs with other modalities, such as immune checkpoint inhibitors and traditional chemotherapy. Combinatorial approaches may not only enhance efficacy but also provide opportunities to modulate the immune response, potentially reducing the risk of adverse events. The synergy between different therapeutic modalities represents a strategic approach to tackling the complexity and heterogeneity of cancer. Furthermore, the development of next-generation BsAbs with extended half-lives and improved pharmacokinetic profiles is contributing to the evolution of these therapies. Enhanced stability and prolonged circulation in the bloodstream may allow for less frequent dosing, minimizing the burden on patients while maintaining therapeutic efficacy. This advancement aligns with the broader trend in oncology towards personalized and patient-friendly treatment regimens. The incorporation of bispecific T-cell engagers and dual-affinity retargeting platforms is another notable development aimed at refining the precision and safety of BsAbs. These platforms leverage advanced engineering strategies to optimize the binding affinity and selectivity of BsAbs, ensuring a more targeted and controlled immune response against tumor cells. Moreover, ongoing research efforts are dedicated to uncovering the intricacies of the tumor microenvironment and its influence on BsAbs efficacy and safety. Understanding the dynamic interplay between immune cells, stromal components, and tumor cells is essential for designing therapeutic strategies that can navigate the complexities of the tumor microenvironment and maximize treatment outcomes.

In brief, the prospects for the safety of BsAbs in the treatment of tumors represent a transformative and promising frontier in cancer therapeutics. The dual targeting mechanism of BsAbs offers a unique opportunity to harness the immune system for precise and potent antitumor responses. Our analysis revealed the tolerable incidence rate of tr-AEs associated with BsAbs compared with chemotherapy monotherapy, providing a solid foundation for the continued exploration of these innovative therapies. As these therapeutic agents progress through clinical trials and gain regulatory approval, the integration of BsAbs into standard cancer treatment protocols holds the potential to redefine the way we approach and combat various malignancies. This paradigm shift offers renewed hope for improved patient outcomes, reflecting the continuous evolution and innovation in the field of cancer therapeutics.

## Ethical approval

Not applicable.

## Consent

Not applicable.

## Source of funding

This work was supported by Postdoctoral Fellowship Program of CPSF (No. GZB20230481), National Natural Science Foundation of China (No. 82303773, No. 82303772, No. 82303694), Natural Science Foundation of Sichuan Province (No. 2023NSFSC1885), Key Research and Development Program of Sichuan Province (No. 23ZDYF2836).

## Author contribution

G.L.: data curation, formal analysis, investigation, writing – original draft, and writing – review and editing; X.S.: data curation, formal analysis, investigation, and writing –original draft; K.K.: formal analysis, investigation, writing – original draft, and writing – review and editing; A.Z.: formal analysis, investigation, supervision, and writing – review and editing; Y.W.: data curation, formal analysis, investigation, supervision, and writing – review and editing.

## Conflicts of interest disclosure

Not applicable.

## Research registration unique identifying number (UIN)

Not applicable.

## Guarantor

Dr Yijun Wu.

## Data availability statement

The data used in the article are shown in Table 1.

## Provenance and peer review

Not applicable.

## Supplementary Material

**Figure:**